# Examining resilience of individuals living with sickle cell disease in the COVID-19 pandemic

**DOI:** 10.1186/s40359-022-00862-0

**Published:** 2022-06-20

**Authors:** Ashley J. Buscetta, Khadijah E. Abdallah, K. Jameson Floyd, Faeben S. Wossenseged, Corinne A. Conn, Hasmin C. Ramirez, Vence L. Bonham

**Affiliations:** 1 Social and Behavioral Research Branch, National Human Genome Research Institute, National Institutes of Health, Bethesda, Maryland USA; 2 Emory University, Atlanta, Georgia USA; 3 University of California San Francisco School of Medicine, CA San Francisco, USA

**Keywords:** Sickle cell disease, Psychological resilience, COVID-19, Psychosocial factors

## Abstract

**Background:**

The COVID-19 pandemic has impacted the physical and mental health of people worldwide including those living with genetic conditions. Sickle cell disease (SCD) is a hematologic chronic disease that causes multisystem damage and morbidity. Individuals living with SCD have had to continue managing their care for their chronic disease while following public health measures to protect against infection with COVID-19. Promoting resilience has been posited as being psychologically protective for those living with SCD. This study examines changes in resilience over time in a SCD population in the context of the COVID-19 pandemic.

**Methods:**

Ninety-seven adults living with SCD completed two parent studies: (1) The INSIGHTS Study, a cross-sectional natural history study conducted from 2014–2019 and (2) The Living with SCD in COVID-19 Pandemic Study, an online survey conducted in 2020. Changes over time in resilience, perceived stress, emotional distress, and physical and mental health were analyzed in multivariable repeated measures model.

**Results:**

Results showed that the psychological resilience of our study cohort had significantly decreased (0.19, p=0.01) over time. Resilience during the pandemic was associated with better mental health and physical health and lower perceived stress and emotional distress. In addition, results showed that marital status, education level, and employment were significantly associated with the psychological resilience of study participants.

**Conclusion:**

Resilience declined during the COVID-19 pandemic but was still associated with better physical and mental health outcomes. Future studies should investigate the relationship between resilience and sociodemographic factors.

In the beginning of 2020, the world experienced exponentially increasing rates of infection by a coronavirus known as SARS-CoV-2, which is a highly infectious and potentially lethal virus that causes COVID-19 [1]. On March 11, 2020, the World Health Organization (WHO) declared that COVID-19 was a global pandemic [2]. In the two years since the pandemic was declared, the United States has suffered some of the worst outcomes across the globe. The United States had the highest global confirmed case count between June and December of 2020, with confirmed case counts increasing from nearly 1.8 million to over 18 million during that time period [3]. Confirmed coronavirus deaths were also some of the highest globally in the United States during this time period. Between June and December of 2020, confirmed deaths increased from roughly 100,000 to nearly 330,000 in number [3] .

COVID-19 is a novel coronavirus that was never before seen in humans and was the first coronavirus to spark a pandemic [4]. Its novelty meant that feelings of uncertainty and fear would persist in the public as the scientific community raced to study this new virus and develop prevention and treatment options. Without a full understanding of COVID-19’s infectivity, transmissibility, and virology, people were reliant on simple public health measures, such as wearing masks and physical distancing, to protect their health.

As scientific knowledge of COVID-19 increased, it became apparent that certain populations, such as those living with sickle cell disease (SCD), may be at higher risk of suffering from a severe disease course. Sickle cell disease is the most commonly inherited hematologic genetic disorder in the United States. It affects approximately 100,000 people in the United States (U.S.), most of whom are of African descent [5] [6]. It is caused by a single point mutation in the hemoglobin beta chain found on chromosome 11 which produces Hemoglobin S (HbS). SCD is a chronic disease with potentially devastating acute complications, such as acute chest syndrome, vaso-occlusion, and infection [7]. In the first two months after the WHO declared COVID-19 to be a pandemic, Panepinto et al. (2020) performed an analysis of persons (n=178) living with sickle cell disease and COVID-19 in the United States. Data was collected by the SECURE-SCD Registry that was established by the Medical College of Wisconsin. The study found that 69% were hospitalized, 11% required ICU admission, and 7% died [8]. The Centers for Disease Control (CDC) deemed SCD to be an underlying medical condition that increases a person’s risk of severe illness from COVID-19 [9]. Impaired immunity and cardiopulmonary dysfunction put individuals living with SCD at higher risk for more severe COVID-19 [10, 11]. The latest data from the SECURE-SCD registry (January 14, 2022) reports 973 cases and 19 deaths [12].

The uncertainty and fear surrounding the COVID-19 pandemic has led to a rise in emotional distress, leading to a global mental health crisis among the general population and particularly among groups deemed high risk [13]. Chronically ill populations have especially felt the psychosocial effects of the pandemic, with one study reporting higher levels of anxiety and depression among individuals with chronic diseases [14]. This phenomenon can possibly be attributed to awareness of vulnerability to COVID-19 and decreased access to healthcare, along with isolation and stress exacerbated by quarantine measures [14].

Research regarding the psychosocial impact of COVID-19 on adults living with SCD is limited, however, increased levels of anxiety among pediatric patients have been reported [15]. In general, sickle cell disease has been associated with worsened mental health [16]; literature has demonstrated an association between increased pain episodes among SCD patients and higher levels of anxiety, depression, and emotional distress [17]. Throughout the COVID-19 pandemic, many people living with sickle cell disease are dealing with multiple factors that increase their vulnerability towards worsened mental health; they are living with a chronic disease, can be immunocompromised, and the generally heightened levels of stress and isolation are likely pressuring their strategies for managing emotional well-being. It is also understood that psychological forms of stress are related to vaso-occlusive pain episodes amongst people living with SCD [18]. All of these factors complicate how they access healthcare for their existing SCD. It is important to understand how these myriad stressors are impacting the psychological resilience of individuals living with SCD to better address their mental health during this time. Therefore, studying and addressing the psychological resilience of the SCD population can also benefit their physical well-being.

Research in resilience posits a unique opportunity to expand current understandings on how to improve quality of life and clinical outcomes for patients living with SCD. Resilience has differing meanings as it has been studied in various disciplines using a range of methodologies. However, its role in health and well-being is well recognized by both the European Psychiatric Association (EPA) and the American Psychological Association (APA), becoming a key part of the World Health Organization’s European policy framework– Health 2020—and the United Nations Sustainable Development Goals [19]. The American Psychological Association defines resilience as the process of adapting well in the face of adversity, trauma, tragedy, threats, or significant sources of stress [20]. Others have defined resilience as the power to overcome a shock or crisis and return to an original state [21], or simply the ability to bounce back [22]. Resilience has also been conceptualized as a psychosocial capability to reduce negative emotions while enhancing adaptation during a crisis [23]. Some have posited that resilience is a dynamic interaction of internal and external risk factors and protective factors during a crisis [24]. For the purposes of our study, we utilize Smith et al.'s BRS definition of resilience which states that, broadly, "resilience is the ability to bounce back or recover from stress.”

It has been well documented that the COVID-19 pandemic has impacted the psychosocial health, in particular, resilience, of many people worldwide [25, 26]. However, there are limited studies that specifically measure changes in resilience within a population living with chronic disease, including sickle cell disease, during the pandemic. This is the first study, to our knowledge, to measure changes in resilience over time in a SCD population during the COVID-19 pandemic. One study has demonstrated a relationship between resilience, chronic disease, and depressive symptoms [27]. Both resilience and chronic disease were found to be significant predictors for depressive symptoms during the initial outbreak of COVID-19 [26]. Another study found that those living with a chronic disease are more likely to report higher levels of stress, anxiety, and depression than those living without a chronic disease, and that promoting psychological interventions to help individuals cope and recover from stressful situations is necessary [28]. Consistent with past resilience literature, recent studies focusing on the mental health of chronic disease patients recognize the need for interventions that improve psychological resilience within these populations [29]. Furthermore, there has been a recent call to devote scientific attention to mitigating the impacts of the social threat caused by COVID-19 for communities experiencing chronic pain, amplifying the importance of assessing the resilience of adults living with SCD [30].

This study is a preliminary longitudinal exploration on resilience, perceived stress, emotional distress, and general physical and mental health among a cohort of adults living with SCD in the U.S. In this study, there are two aims: 1) to collect and analyze psychosocial data of adults (18 years or older) living with SCD and 2) to explore factors which influence psychological resilience. In order to investigate the impact that the COVID-19 pandemic had on psychological and physiological well-being, we analyzed data from this cohort collected at two different time points: pre-pandemic and during pandemic.

## Methods

Data used in this analysis was extracted from two parent studies collected between 2014–2020. Participants included in this analysis completed both “pre” and “during” pandemic studies, were age 18 or older, had a diagnosis of SCD including all genotypes, and were current residents in the United States.

### Study sample

#### The INSIGHTS Study

INSIGHTS into the Microbiome and Environmental Contributions to Sickle Cell Disease and Leg Ulcers Study (NCT02156102, registration date 05/06/2014) is an ongoing prospective, cross-sectional, multi-site research study. Data used for this analysis were collected between 2014 and 2019. Surveys were interviewer-administered. Participants for this study either enrolled at the NIH Clinical Center (Bethesda, MD) or the Montefiore Medical Center (Bronx, NY) and were consented and compensated for their time and effort. Study approval was obtained through the National Institutes of Health (NIH) Institutional Review Board (IRB) for protocol number 14HG0125. Data obtained from this study was considered ‘Pre-pandemic’ given the time point.

#### The COVID-19 Sickle Cell Study Survey

Living with Sickle Cell Disease in the COVID-19 Pandemic (NCT04417673, registration date 05/06/2020) is a longitudinal study aimed at understanding the extent and impact of life changes induced by the COVID-19 pandemic on individuals living with SCD in the United States. The study was developed the month following the declaration of COVID-19 as a pandemic in April–May 2020 as an online, self-administered survey instrument, with initial data collection beginning June 2020. Given the longitudinal nature of the study, participants will be interviewed at regular time intervals through 2022. Participants were recruited from the INSIGHTS study. All eligible individuals agreed to participate in the study prior to data collection and were compensated with a $20 electronic gift card upon completion. Study approval was obtained through the NIH Institutional Review Board for protocol number 20HGN125. The need for informed consent was waived by the NIH IRB. Data collected between June 2020 and December 2020 was used for our analysis and was categorized as ‘During pandemic’.

### Primary outcome measures

#### Brief Resilience Scale

The Brief Resilience Scale (BRS) was created by Smith and colleagues at the University of New Mexico in 2008 to assess resilience as the ability to bounce back [22]. Scores are categorized as “low resilience” (1.00–2.99), “normal resilience” (3.00–4.30), and “high resilience” (4.31–5.00). The BRS is considered a good measure of positive correlations with general well-being, social support, and positive reframing of events [31]. In instances where the BRS score was not categorized, higher scores indicate more resilience.

#### Cohen Perceived Stress Scale

The Cohen Perceived Stress Scale (PSS) was coined in 1983 by Sheldon Cohen and is designed to measure the degree to which a respondent considers events over the last month in their life as stressful [32]. A respondent’s daily experiences, major life events, and/or access to coping resources may account for different levels of stress when compared to another respondent experiencing the same event. Scores are categorized as “low stress” (0–13), “moderate stress” (14–26), and “high stress” (27–40).

#### ASCQ-Me Emotional Impact Scale

The Adult Sickle Cell Quality of Life Measurement Information System (ASCQ-Me) Emotional Impact Scale was created in 2005 in a collaboration between the National Heart, Lung, and Blood Institute (NHLBI) and the American Institutes for Research (AIR). The Emotional Impact Subscale consists of five questions on participants’ emotional distress within the past seven day to which respondents answer on a scale of never/not at all (score = 1) to always/very (score = 5), with higher scores indicating better health-related quality of life (QOL) [33] [34].

#### PROMIS Global Mental and Physical Health Scales

The Patient-Reported Outcomes Measurement Information System (PROMIS) was created in 2004 by a cooperative group led by NIH and several U.S. academic institutions [35]. The Global Mental Health (GMH-2) and Global Physical Health (GPH-2) Scales are both 2 item questionnaires with five-category response scales [36]. These measures assess function and well-being within the mental and physical domains of health.

### Statistical analysis

Participant demographics and collected study measurements were summarized using common descriptive statistics. Frequency percentages were reported for categorical variables and mean and standard deviation (SD) for the continuous variables. The Spearman correlation coefficient was used to examine associations between resilience and measures of self-reported health and well-being. Additionally, Student’s paired *t*-tests were utilized to examine the unadjusted mean score differences before and during the COVID-19 pandemic for the following measures: self-reported physical, mental and emotional health, Brief Resilience Scale, and Cohen’s Perceived Stress Scale.

These outcomes (physical health, mental health, emotional health, and Cohen PSS) were then examined in a repeated measures model to assess change over time in relation to resilience. The multivariable repeated measures models adjusted for sociodemographic and clinical variables including age (young [18–40 years] vs. older [40+ years]), sex (male vs. female), currently married (no vs. yes), currently employed (no vs. yes), current educational level (high school degree or some college vs. a bachelor’s degree or higher), and initial SCD clinical severity (low vs. high severity) [37]. Additionally, we were interested in within-subject changes for the resilience categories pre- and during pandemic, so an interaction term was included to examine the time effect of resilience on the outcomes. Regression diagnostics, using studentized residual analysis and a cut-off range between — 2 and 2, were performed to identify and subsequently remove outliers. Statistical significance for all results were determined based on a p-value of <0.05. All statistical analyses were completed using SAS version 9.4.

## Results

Ninety-seven participants (mean age= 47.3, SD= 11.2) were included in this study. Demographic variables can be found in Table 1. The majority of the population was female (63%) and identified as Black/African American (95%). Approximately, 45% had a bachelor’s degree or higher, 47% were currently working, and 41% were married or living with a partner. Income levels of $60,000 and higher were reported by 37% of the study population.

**Table 1:** Descriptive characteristics of pre-pandemic and during pandemic participants

Variable	Pre-pandemic		During pandemic	
	N = 97	%	N = 97	%
**Age**, mean (SD/range)	39 (11.2, 19–66 years)	–	41.3 (11.2, 21–68 years)	–
**Sickle cell disease severity**				
High severity	–	–	28	28.9
Low severity	–	–	69	71.1
**Sex**				
Male	37	38.14	36	37.1
Female	60	61.86	61	62.9
**Race/ethnicity**				
Black/African American^a^	93	96.9	89	94.7
Hispanic^b^	6	6.3	6	6.5
**Insurance status**				
Private^c^	49	51	44	45.4
Medicare/medicaid/Medigap/SCHIP	33	34.4	34	35.1
Government health insurance^d^	4	4.2	7	7.2
No coverage	10	10.4	–	–
**Education**				
Some college and below^e^	47	48.5	37	38.1
Bachelor’s/master’s degree	44	45.4	44	45.3
Professional/doctoral degree	6	6.2	5	5.2
**Marital status**				
Married/living with partner	30	30.9	40	41.2
Divorced/separated/widowed	13	13.4	12	12.4
Never married	54	55.7	45	46.4
**Income**				
Less than $10k - $29k	34	36.6	28	29.8
$30k - $59k	29	31.2	31	33
$60k - $90k	13	14	13	13.8
Greater than $90k	17	18.3	22	23.4
**Employment status**				
Working currently	46	47.4	46	47.4
Retired/student/keeping house	14	14.4	12	12.4
Unemployed/looking for work/lost job	19	19.6	8	8.2
Disabled	18	18.6	23	23.7
Other	–	–	7	7.2

^a^Black/African American vs. American Indian/Alaska Native, Asian, Native Hawaiian/Other Pacific Islander, White

^b^Hispanic/Latino vs. Non-Hispanic/Latino

^c^Private: private health insurance, employer health insurance, private + other government insurance

^d^Government Health Insurance: Military Health Care, Indian Health Service, government health insurance marketplace plan, other government plan

^e^Some College and Below: eighth grade or below, high school graduate or equivalent, some college, associate degree

**Fig 1:**
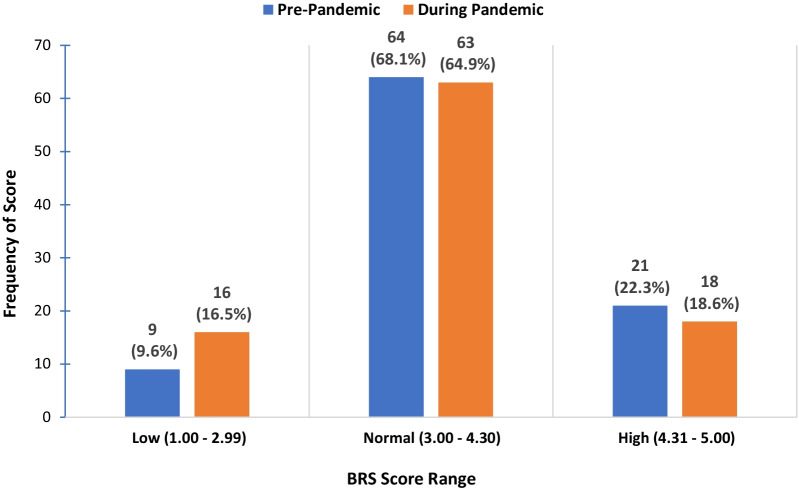
Brief resilience score frequency pre/during pandemic

Resilience scores decreased by an average of 0.19 points (p=0.01) during the COVID-19 pandemic in comparison to pre-pandemic (Table 2). Furthermore, there was a 7% increase in the number of participants with a BRS score below 2.99, a range categorized as “Low” resilience (Fig 1). Participants reported incidence of COVID-19 infection and possible exposure during the two weeks prior to survey completion. (Table 3). Overall, a majority of study participants denied being diagnosed with (n=91) or exposed to (n=93) COVID-19. Ninety-two participants also denied having any family members diagnosed with COVID-19. Slightly over half of the participants (n=51, 53%) reported having at least one symptom that was associated with COVID-19 infection, such as fever, cough, or shortness of breath.

**Table 2:** Mean psychosocial scale scores during pandemic and mean score differences pre and during pandemic

Psychosocial scale	Mean*	SD	Range	Mean difference	*P* value
Brief Resilience Scale	3.6	0.78	1.0–5.0	0.195	0.01
ASCQ-Me Emotional Impact Scale	10.4	4.5	5.0–25.0	0.075	0.88
Cohen’s Perceived Stress Scale	19.9	6.4	5.0–20.0	— 1.163	0.07
PROMIS Global Mental Health Scale	13.4	3.3	7.0–20.0	0.448	0.09
PROMIS Global Physical Health Scale	12.6	2.8	7.0–18.0	0.24	0.35

*Mean score during pandemic

**Table 3:** Incidence of COVID-19 related fear, threat, worry, infection, and exposure

Variable	N	%
**Fear of COVID-19**		
Not at all	17	17.5
A little bit	19	19.6
Somewhat	15	15.5
Very much	24	24.7
Extremely	22	22.7
**Feeling threatened by COVID-19**		
Not at all	16	16.5
A little bit	20	20.6
Somewhat	26	26.8
Very much	25	25.8
Extremely	10	10.3
**Worried about COVID-19 Infection**		
Not at all	18	18.6
Slightly	24	24.7
Moderately	20	20.6
Very	17	17.5
Extremely	18	18.6
**During the past two weeks, have you been exposed to someone likely to have Coronavirus/COVID-19?**		
Yes^a^	4	4.1
No	93	95.9
**During the past two weeks, have you been suspected of having Coronavirus/COVID-19 infection?**		
Yes^b^	6	6.2
No	91	93.8
**During the past two weeks, has anyone in your family been diagnosed with Coronavirus/COVID-19?**		
Yes^c^	6	6.2
No	92	93.8
**During the past two weeks, have you had any of the following symptoms?**		
Fever	6	6.2
Cough	15	15.5
Shortness of breath	12	12.4
Sore throat	6	6.2
Fatigue	35	36.1
Loss of taste or smell	2	2.1
Other symptom^d^	52	53.6

^a^Yes, someone with a positive test; Yes, someone with a medical diagnosis, but no test; Yes, someone with possible symptoms, but no diagnosis by a doctor

^b^Yes, I have had a positive test; Yes, I have been tested and tested negative; Yes, I had a medical diagnosis, but no test; Yes, have had some possible symptoms, but no diagnosis by doctor

^c^Yes, member of household; Yes, non-household member

^d^Other: runny nose, sneezing, muscle/body aches, vomiting, diarrhea, other symptom

Spearman correlation coefficient values for “during pandemic” resilience scores in relation to the outcomes are demonstrated in Table 4. Current resilience levels were significantly associated with less worry about being infected (rho= — 0.31, p<0.0001), less emotional distress (rho= — 0.47, p<0.0001), less perceived stress (rho= — 0.55, p<0.0001), and feeling less threatened (rho — 0.34, p<0.001) by COVID-19. Current resilience values were also significantly associated with higher mental health scores (rho= 0.48, p<0.0001) and physical health scores (rho= 0.25, p=0.01) (Table 4).

**Table 4:** Spearman correlations for Brief Resilience Scale during COVID-19 pandemic

Measure	Resilience	
	rho	*p* value
Mental health	0.48	<.0001
Emotional distress	— 0.47	<.0001
Physical health	0.25	. 01
Perceived stress	— 0.55	<.0001
Worried about COVID-19 Infection	— 0.31	.002
Threatened by COVID-19	— 0.32	.002
Fear of COVID-19	— 0.18	.08

Table 5 presents the results of the longitudinal analysis for the five outcomes. The time effect was only significant for the resilience outcome, such that the observed decrease in resilience levels over time remained significant even after adjusting for sociodemographic and clinical variables (p<0.01). Unemployment status (β= — 0.28, p<.05), not being married (β= — 0.33, p<.01), and having lower educational attainment defined as high school degree or some college (β = — 0.41, p<.01) were significantly associated with lower resilience levels, after adjusting for covariates. For other outcomes, high resilience, compared to normal resilience, was significantly associated with higher (positive) mental health scores (β= 1.77, p<.05), as well as lower emotional distress (β= — 2.22, p<.05) and lower perceived stress (β= — 5.51, p<.01). Employment status was the only factor that was significantly predictive of all the outcomes with those reporting current unemployment experiencing lower mental health scores (β= — 0.81, p<.001), lower physical health scores (β= — 0.81, p<.001), higher emotional distress (β= — 1.75, p<.001), and higher perceived stress (β= 3.15, p<.001).

**Table 5:** Repeated measures models of predictors and health outcomes

Variable	Model^a,b^: outcomes and parameters estimates				
	Mental health	Emotional impact/distress	Physical health	Perceived stress	Resilience
**Sex**					
Female vs. Male	— 0.35	0.31	— 0.44	1.4	0.07
**SCD clinical severity**					
Low vs. High	0.23	— 0.42	0.24	0.67	— 0.26
**Employment status**					
Unemployed vs. employed	— 0.81**	2.09**	— 1.75**	3.15**	— 0.28*
**Married**					
No vs. Yes	— 0.76	1.42	0.19	0.83	— 0.33**
**Educational status**					
Highschool or some college vs. Bachelor’s or higher	— 1.43*	2.01**	— 1.13*	1.7	— 0.41**
**Age category**					
Younger vs. older	— 0.37	0.07	0.91	2.09*	0.001
**Resilience category**					
High resilience vs. normal	1.77*	— 2.22*	0.33	— 5.51**	–
Low resilience vs. normal	— 0.59	1.81	— 0.42	0.86	–
**Timepoint**					
Pre- vs. during pandemic	0.52	0.48	0.09	— 1.23	0.21**
**Resilience category timepoint **^**b**^					
High pre-pandemic vs. during pandemic	— 0.56	0.18	0.13	0.43	–
Low pre-pandemic vs. during pandemic	— 0.24	— 0.66	1.3	0.44	–

*p<0.05, **p<0.01, ***p<0.001

^a^Parameter estimates or the β coefficient, give the difference in the mean scores for the outcomes (Mental Health, Emotional Impact/Distress, Physical Health, Perceived Stress, Resilience) between the indicated group and reference group (e.g., Female vs. Male or Not Working vs. Working).

^b^The interaction terms were only tested for time and resilience to look at the effect of time on within group differences for the resilience categories and their association with the outcomes.

## Discussion

To our knowledge, this is the first study to examine longitudinal resilience in a sickle cell disease adult population during the COVID-19 pandemic. There was a significant decrease in resilience levels during the pandemic. Over time, this cohort also experienced increased perceived stress with a decline in physical health, mental health, and emotional distress, but these were not statistically significant. Despite the overall decline in resilience over time, the level of resilience that remained during the pandemic was psychosocially protective, defined as “assets and resources within the individual, their life, and environment that facilitate the capacity for adaptation and ‘bouncing back’ in the face of adversity” [38]. Lupe et al. (2020) showed that people with lower resilience can gain the ability to adapt to adversity [39]. The “during pandemic” resilience levels were associated with feeling less threatened by COVID-19 and worrying less about becoming infected, indicating some degree of its psychosocial protectiveness.

In this cohort, very few people reported exposure to or infection with COVID-19, but over half reported possible COVID-19 associated symptoms at the time of survey. This kind of uncertainty about one’s health status, as well as the lack of testing, knowledge, and misinformation that plagued the beginning of this pandemic, may have contributed to psychological distress. The COVID-19 pandemic is responsible for causing stress, trauma, fear, and feelings of uncertainty for the general population [13]. Overall, a majority of this cohort reported feelings of fear (82.5%), threat (83.5%), and worry (81.4%) about COVID-19. Looking closer at those with higher resilience (>4.31) showed that this subgroup had better mental health, less emotional distress, and lower perceived stress, along with worrying less, feeling less threatened, and experiencing less fear from COVID-19. Those with lower resilience experienced a decline in physical health over time compared to those with normal or high resilience whose physical health remained steady.

Some socioeconomic factors were significantly associated with resilience. Those with lower resilience were more likely to be unemployed, unmarried, and less educated. Employment status was a significant predictor for mental and physical health, emotional distress, and perceived stress. This is consistent with the literature and important to note as less than half of the cohort reported they were currently working. Knisely et al. (2020) showed that unemployment in a SCD population was significantly associated with worsening pain impact and social functioning [40]. Nationwide, there was a record-breaking unemployment rate (13% of U.S. population) in the first 3 months of the pandemic [41]. Unemployment directly related to the pandemic compounded the stress of the situation. Loss of one’s job and income can contribute to a lack of feeling in control and to lower self-worth, which can lead to worsening mental and emotional health [42]. Marital status was significantly associated with resilience scores. There was a marginal (10%) increase in those reporting to be married or living with a partner during COVID. The role of a partner or spouse can be a significant source of caring and support for any adult living with a chronic disease, helping to increase coping and mitigate stress, and thus increase resilience [43]. Lower educational attainment is associated with chronic health conditions, which may explain why nearly half of participants in this cohort report having a college degree or lower [44]. In general, connections between education and health and emotional well-being are present across multiple populations, making the association between resilience and education an expected finding [45–47].

People at higher risk for COVID-19 disease may experience adverse psychosocial health outcomes. Prior stress and resilience research has shown the higher the controllability of a stressful situation is, the better individuals cope with this situation [48]. Sickle cell disease is inherently unpredictable and similar to stress in terms of its dynamic nature and severity [18]. It is a chronic disease with acute complications, which often leaves those affected experiencing a lack of control over their own body and health. It is important for those living with SCD to be equipped with positive coping mechanisms in response to their highly variable chronic disease [49].

## Limitations

There are a few limitations to account for within this study. First, the sample size was small, limiting the generalizability and representativeness of this study. Additionally, survey administration differed between the two parent studies. The INSIGHTS study survey was interviewer-administered and the COVID SCD survey was self-administered. Respondents may have also been susceptible to social desirability bias in order to appear more aligned with recommended health measures. Self-reported psychological measures may also not be perfectly aligned with assessments by mental health professionals [27]. The vagueness and lack of specificity with most of the associated COVID-19 symptoms coupled with decreased access to healthcare and diagnostic testing to detect SARS-CoV-2 could create a generalized state of anxiety. Also, we did not assess the quality of the demographic factors our participants reported at the time of our COVID survey. For example, it is well documented that during the pandemic there has been a rise in interpersonal violence within partnerships and families [50]. As we were not able to assess the healthiness of this demographic factor, there is risk of misattributing marriage as automatically relating to higher levels of resilience in our participants. Additionally, further research is needed to better understand how unemployment and education have contributed to the overall decline in resilience observed during the pandemic. We suggest a qualitative approach would be helpful to describing what precisely impacts the resilience of our population, particularly during these unprecedented times.

## Conclusion

The COVID-19 pandemic has exacerbated existing health disparities for vulnerable populations in the U.S. [51]. Our study investigated the relationship between the mental and physical well-being of adults living with SCD during a pandemic. While our study reinforces existing literature demonstrating that resilience, as a psychological capacity to bounce back from stress, is related to other indicators of physical and mental health, it also demonstrates that resilience is a psychologically and physically protective resource in this population during the specific stress created by the pandemic. Thus, those individuals who indicated higher levels of resilience prior to the pandemic continued to have better physical and mental health outcomes throughout the period of data collection. This emphasizes the need to build resiliency throughout the lives of individuals, prior to a traumatic event, for its protective capacities to impact health outcomes. Utilizing the Brief Resilience Scale in clinical encounters to screen those with low resilience may lead to implementing helpful interventions to prevent some of the adverse physical and psychosocial outcomes associated with lower levels of resilience. It also posits that collecting information on resilience over time may have clinical utility in the lives of individuals living with chronic illness, particularly as a tool to coordinate preventive care.

In our cohort, individuals living with SCD were not immune to the stress of COVID-19, as resilience scores waned in relation to factors directly impacted by the pandemic, such as a rise in unemployment. Vulnerable populations, such as the SCD community, are often portrayed as having “grandeous” amounts of resilience compared to the general population [52]. While financial instability is a known factor related to lessened resilience, SCD populations has historically had to interact with this stressor throughout their lives as a burden of SCD and living with a chronic disease [53]. In addition, our population is more likely to be unemployed or on disability compared to the general population prior to COVID-19 [54]. Therefore, our findings question the impact that these known factors have on a community that lives with an unpredictable disease and historically withstands significant trauma. Additionally, while we advocate for resiliency in times of stress, trauma, and uncertainty, we need to uphold a balanced perspective and investigate the structural factors that are at the root of the problems faced by this population. The resiliency in our study participants coupled with their lived experiences of dealing with SCD did not provide them with a unique ability or advantage to live in a world with COVID-19. As they were still susceptible to the overall stress caused by the pandemic, further research is needed to better understand how to protect this community from forces that lessen their resilience. As this study is the first to look at resilience over time in a population of adults living with SCD, we hope this provides invaluable insights for clinicians and researchers into the psychological well-being of people living with SCD.

## Data Availability

The datasets generated and analyzed during the current study are available in the openICPSR repository, https://doi.org/10.3886/E166321V1

## Abbreviations

AIR: American institutes for research
APA: American psychological association
ASCQ-Me: Adult sickle cell quality of life measurement information system
BRS: Brief resilience scale
CDC: Centers for disease control
EPA: European psychiatric association
IRB: Institutional review board
NHLBI: National heart, lung, and blood institute
NIH: National institutes of health
PROMIS: Patient-reported outcomes measurement information system
PSS: Perceived stress scale
QOL: Quality of life
SCD: Sickle cell disease
SD: Standard deviation
